# The Possible Application of Ketamine in the Treatment of Depression in Alzheimer’s Disease

**DOI:** 10.3390/neurolint14020025

**Published:** 2022-03-22

**Authors:** Islam Mohammad Shehata, Waniyah Masood, Nouran Nemr, Alexandra Anderson, Kamal Bhusal, Amber N. Edinoff, Elyse M. Cornett, Adam M. Kaye, Alan D. Kaye

**Affiliations:** 1 Anesthesia Department, Faculty of Medicine, Ain Shams University, Cairo 11517, Egypt; islam.shehata@med.asu.edu.eg; 2 Department of Medicine, Dow Medical College, Dow University of Health Sciences, Karachi 74200, Pakistan; waniyah.masood17@dmc.duhs.edu.pk; 3 ICU and Pain Management, Faculty of Medicine, Ain Shams University, Cairo 11517, Egypt; nourannemr95@gmail.com; 4 Department of Medicine, LSU Health Shreveport, 1501 Kings Hwy, Shreveport, LA 71103, USA; aia002@lsuhs.edu (A.A.); kamal.bhusal@lsuhs.edu (K.B.); 5 Department of Psychiatry and Behavioral Medicine, LSU Health Shreveport, 1501 Kings Hwy, Shreveport, LA 71103, USA; 6 Department of Anesthesiology, LSU Health Shreveport, 1501 Kings Hwy, Shreveport, LA 71103, USA; elyse.bradley@lsuhs.edu (E.M.C.); alan.kaye@lsuhs.edu (A.D.K.); 7 Department of Pharmacy Practice, Thomas J. Long School of Pharmacy and Health Sciences, University of the Pacific, Stockton, CA 95211, USA; akaye@pacific.edu

**Keywords:** Alzheimer’s disease, dementia, depression, ketamine, NDMA antagonists, esketamine

## Abstract

Depression is a leading cause of disability globally, with a prevalence of 3.8% among the whole population, 5% of the adult population, and 5.7% of the elderly population over 60 years of age. There is evidence that depression is linked to certain neurodegenerative diseases, one being Alzheimer’s disease (AD). The efficacy of conventional antidepressants to treat depression in AD is conflicting, especially regarding selective serotonin reuptake inhibitors (SSRIs). A recent systemic review and meta-analysis of 25 randomized controlled trials including fourteen antidepressant medications showed no high efficacy in treating AD patients’ symptoms. However, ketamine, a nonselective N-methyl-D-aspartate (NMDA) receptor antagonist, can mediate a wide range of pharmacological effects, including neuroprotection, anti-inflammatory and anticancer properties, multimodal analgesia, and treatment of depression, suicidal attempts, and status epilepticus. Esketamine, which is ketamine formulated as a nasal spray, was approved by the Federal Drug Administration (FDA) in March 2019 as an adjuvant drug to treat treatment-resistant depression. NMDA receptor antagonists treat AD through offsetting AD-related pathological stimulation of subtypes of glutamate receptors in the central nervous system. Recent clinical findings suggest that ketamine may provide neuroprotection and reduce neuropsychiatric symptoms associated with AD. In the present investigation, we evaluate the potential role of ketamine and its postulated mechanism in AD management.

## 1. Introduction

Depression is a leading cause of disability globally, with a prevalence of 3.8% among the whole population, 5% of the adult population, and 5.7% of the elderly population above 60 years of age [1]. It is characterized by a lack of desire to engage in normal activities or previously desirable activities, persistent sadness, or irritation affecting one’s social and professional life [2]. There is evidence that depression is linked to some neurodegenerative diseases; one is Alzheimer’s disease (AD) [2,3]. AD is characterized by memory loss, decreased cognitive abilities, decreased visuospatial skills, and personality change. These pathological features are attributed to the accumulation of β-Amyloid (Aβ), which stimulates an inflammatory response causing neuronal damage [4,5]. Depression is reported in 30–40% of patients suffering from AD [6].

Ketamine, a nonselective N-methyl-D-aspartate (NMDA) receptor antagonist, can be used to treat depression [7]. A single sub-anesthetic dose of ketamine can be an alternative to electroconvulsive therapy in treatment-resistant depression (TRD) given its rapid action [8]. Moreover, ketamine has a rapid and prolonged antidepressant effect compared to currently approved antidepressants [9,10]. These studies suggest that ketamine may be an option in treating AD-related depression [11]. Esketamine, which is ketamine formulated as a nasal spray, was approved by the Federal Drug Administration (FDA) in the United States in March 2019 as an adjuvant drug in the treatment of TRD [12].

The unclear role regarding the use of ketamine as a potential therapy for depression in AD is due to ketamine’s side effects of dissociation, memory loss, confusion, and the likelihood of abuse. Additionally, the exact mechanism by which ketamine can potentially benefit depression symptoms is largely unknown. At this point in time, clinical studies in the population suffering from both AD and depression are lacking. This review is, therefore, a bit theoretical in nature, but new treatments should be considered. In the present investigation, therefore, we evaluate the potential role of ketamine and its postulated mechanism in the management of AD.

## 2. Alzheimer’s Disease

AD is a progressive neurodegenerative disease that mainly affects older adults. It was estimated that in 2020, about 5.8 million Americans were suffering from AD, and this number is projected to triple by 2060 [13]. Other estimates suggest that the number is around 50 million worldwide and is projected to reach 152 million by 2050 [14].

### 2.1. Pathogenesis

Pathogenesis mainly involves the progressive loss of cortical neurons related to atrophy and classical positive lesions formed by the accumulation of amyloid plaques, neurofibrillary tangles (NFT), dystrophic neuritis, neuropil threads, and other deposits. Amyloid plaques are created by excess production and decreased clearance of Aβ peptides. The major constituent of NFTs is hyperphosphorylated tau protein.

### 2.2. Causes and Risk Factors

The risk factors associated with AD include age, genetic predisposition, head injuries, vascular disease, infections, hypertension, diabetes mellitus, dyslipidemia, medications, and environmental factors (e.g., air pollution, heavy metals, pesticides, etc.). There is some evidence that impairment of cholinergic function is a critical risk factor, but others believe that alteration in Aβ-protein production and processing is the main factor.

### 2.3. Clinical Features

AD is a progressive disease. Life expectancy after diagnosis is about 8–10 years but can vary greatly from 3–20 years [15,16]. Impairment in memory, executive function, judgment, visuospatial ability, language and behavior, and psychological symptoms (apathy, social disengagement, irritability, agitation, aggression, wandering, and psychosis) are the cardinal symptoms of AD. Memory impairment, defined as the specific loss of the memory of a recent event, is an initial and most common symptom [17].

Non-cognitive neurological deficits like seizures, myoclonus, extrapyramidal and pyramidal signs occur in the late stages of AD. Dyspraxia (a problem in learned motor tasks) can occur late in the disease. The disease affects complex activities like dressing and other self-care tasks, making patients dependent on others [18]. Other presentations of AD can be olfactory dysfunction and sleep disturbances. It should be noted that the sleep disturbances are multifactorial in AD. Medications, such as donepezil, used to treat the cognitive symptoms of AD can actually lead to sleep disturbances by altering the patient’s sleep architecture [19]. The use of donepezil can increase REM sleep but also decrease stage 2 sleep percentage, sleep efficiency, and total sleep time [19].

A few patients, mainly those who are younger at presentation, may not present with classic symptoms of progressive amnestic dementia and may present with atypical symptoms like progressive cortical visual impairment, primary progressive aphasia, and deficit in executive function (dysexecutive or frontal variant). It can also coexist with other dementias, including vascular lesions, cortical Lewy bodies, and Parkinson’s disease.

### 2.4. Evaluation

Extensive cognitive and general neurological examination is needed if an elderly patient presents with progressive memory decline or has other cognitive impairments. Other possible causes of dementia should be ruled out. Standardized mental status scales for neuropsychological testing should be used which will help establish a baseline and monitor disease progression in the future.

Imaging testing could help establish a diagnosis, which includes MRI, FDG-PET and SPECT, and the amyloid PET scan. Tau PET imaging is in development. MRI findings can be either focal or generalized atrophy, and there may be no specific white matter lesions. Hippocampal volume and medial temporal lobe atrophy are also common.

Several biomarkers can support the diagnosis, but they are not recommended for routine testing. Molecular biomarkers that indicate Aβ protein deposition include low CSF Aβ42 (or Aβ42:Aβ40 ratio) and a positive PET scan with amyloid PET tracer. Increased CSF total tau and phosphor-tau and Tau PET scan with flortaucipir F-18 are indicators of tau deposition. Genetic testing is reserved for selected cases, especially for young-onset patients.

### 2.5. Diagnosis

Clinically, AD is diagnosed with an insidious onset and progressive cognitive decline in one or more domains. The Diagnostic and Statistical Manual of Mental Disorders 5 (DSM-5) defines these domains as learning and memory, language, executive function, complex attention, perceptual-motor, and social cognition for neurocognitive disorders in general [20]. The scans and biomarkers discussed above help guide the diagnosis, but usually clinical diagnoses. If this decline is seen gradually, AD is probably the cause of the neurocognitive disorder. Definitive diagnosis is completed with histopathology obtained from a brain biopsy, which is rarely done.

It should be noted that many of the same symptoms one may see in depression may also be seen in AD patients without depression. These symptoms can be neurovegetative symptoms such as decreased sleep, poor mood, decreased appetite, and decreased concentration. This makes it hard to at times delineate true depression that is in AD patients or symptoms that are from the AD itself. This is where history and family collateral information comes in handy, and may help the clinician decide if the patient’s symptoms are truly stemming from depression.

## 3. Current Alzheimer’s Treatments and Shortcomings

There have been many challenges in the development of successful therapies for AD. This is because diagnosing AD can be very difficult for clinicians [21]. Evidence suggests that pathological changes can develop in AD decades before individuals begin experiencing symptoms, making certain medications less successful by the point of later diagnosis [21]. Another major challenge exists with risk factor differences between men and women in clinical presentation [21]. Preclinical studies have suggested that menopausal changes may be a risk factor for AD in women [21]. To date, no medications have successfully cured individuals or slowed the progression of AD [21]. All available treatments currently approved by the FDA are designed for supportive care of symptomatic treatment in AD, including behavioral and cognitive deficits [22].

Additionally, there are limited medications available to treat psychiatric symptoms in AD [22]. There are no drugs currently approved to treat psychosis, apathy, or depression in AD [22]. However, the drug pimavanserin is a serotonin receptor antagonist that may soon receive approval by the FDA to treat AD-related psychosis [22]. Standard therapies for major depression have mostly proved unsuccessful in treating depression [22]. AD pathology is very complex, and therefore many standard therapies for other neurological diseases are not effective in treating this disease. The neurobiological basis for these symptoms is still an area of investigation, and if treated, can prevent further decline and behavioral problems in many individuals [22]. Although there are limited options available currently, there are several therapies undergoing clinical trial testing.

Currently, acetylcholinesterase inhibitors and N-Methyl-D-aspartic acid (NMDA) antagonists are the only medications approved for use in AD by the FDA [23]. Acetylcholinesterase inhibitors reduce the breakdown of acetylcholine by the enzyme acetylcholinesterase in the synaptic cleft [23]. These medications are designed to slow the decline in cognition and are most effective when started soon after the time of diagnosis [23]. Galantamine, one acetylcholinesterase inhibitor, is approved for treating mild to moderate AD, while rivastigmine and donepezil can be used in any stage of treatment for AD [23]. Individuals taking these medications may experience symptoms such as nausea, vomiting, and diarrhea [23]. Memantine is an approved medication for AD that works differently from acetylcholinesterase inhibitors by blocking the NMDA receptor and affecting the action of glutamate, which plays a role in memory [24]. Memantine can be used for moderate to severe AD in combination with acetylcholinesterase inhibitors, or on its own [24].

Other treatment mechanisms include gamma-secretase inhibitors and alpha-secretase modulators, which prevent the cleavage of amyloid precursor proteins into the misfolded dysfunctional protein product that accumulates in AD [23]. Currently, there are several active clinical trials focusing on Aβ immunotherapy [23]. Results from these clinical trials have even demonstrated that participants in the trials can produce specific antibodies against the C-terminal end of Aβ 40 [23]. Some of the most promising research has been in passive Aβ immunotherapy [23].

Aducanumab is a specific monoclonal antibody given through intravenous infusion and has been designed to target Aβ [25]. This medication has been approved by the FDA for treating patients with mild cognitive deficits and mild AD because it decreases Aβ accumulation in the brain, which has been measured in trials with PET imaging [25]. Individuals receiving this therapy must have regular brain MRIs prior to the initiation of infusion and additional imaging to receive other doses [25]. However, thus far, the clinical benefit is considered relatively insignificant [25]. The drug was approved on the basis that the reduction in Aβ will likely lead to a positive clinical benefit and because there are so few options available for treating AD [25].

## 4. Evidence for Ketamine Treatment

Ketamine was developed in the 1960s as an anesthetic agent, and its neurobehavioral effects have been identified over the last 50 years [26]. Ketamine is a racemic mixture comprising equal parts of (R)-ketamine (or arketamine) and (S)-ketamine (or esketamine). Ketamine, a nonselective N-methyl-D-aspartate (NMDA) receptor antagonist, can mediate a wide range of pharmacological effects, including neuroprotection, anti-inflammatory and anticancer properties, multimodal analgesia, and treatment of depression, suicidal attempts, and status epilepticus [27]. One of the non-competitive NMDA receptor antagonists, memantine, showed therapeutic benefits in AD [28]. The theory behind NMDA receptor antagonists in AD treatment lies in implementing NMDA receptor blocking agents in offsetting Alzheimer-related pathological stimulation of these subtypes of glutamate receptors in the central nervous system [29]. The excitotoxic hypothesis is speculated in the pathogenesis of AD, where the overactivation of glutamate, the primary excitatory amino acid, causes neurotoxicity [30]. Comparing ketamine to memantine, the latter exhibits lower affinity to the NMDA receptors with rapid relief of its block, which allows symptomatic improvement without affecting desirable functions such as memory and learning [5]. However, ketamine showed rapid relief of major depression symptoms in patients with AD [31]. The US FDA and European Medicines Agency recently approved intranasal S-ketamine for treatment-resistant depression in conjunction with an oral antidepressant [32]. At the same time, the data about the efficacy of the conventional antidepressant in AD are still conflicting, especially with regard to the selective serotonin reuptake inhibitor (SSRI) [33]. A recent systemic review and meta-analysis of 25 randomized controlled trials, including fourteen antidepressant medications, did not show high efficacy in the treatment of the symptoms in AD patients [34]. The efficacy of ketamine may be attributed to the diverse postulated antidepressant mechanisms, which include both NMDA-dependent and independent mechanisms [8]. The NMDA receptor inhibition independent mechanisms belong to the ketamine metabolites; (S)-norketamine and (2R,6R)-hydroxynorketamine (HNK) [35]. The proposed mechanisms include enhancement of the synaptic plasticity and activation of brain-derived neurotrophic factor (BDNF), eukaryotic elongation factor 2 (eEF2), mechanistic target of rapamycin (mTOR), and glycogen synthase kinase-3 (GSK-3). A unique advantage of ketamine’s antidepressant action is the rapid and long-lasting effect, especially with (R)-ketamine, which poses less detrimental side effects than (R,S)-ketamine or (S)-ketamine [36]. Moreover, a significant incidence of orthostatic hypotension in this cohort had been reported with the administration of antidepressant therapy, especially with SSRIs [37]. On the other hand, increases in both systolic and diastolic blood pressure as well as heart rate with ketamine administration are evident with short-term intravenous ketamine administration [38]. However, these ketamine-related cardio stimulatory effects are dose-dependent and are seen less with oral ketamine, especially in young and healthy adults [39]. Therefore, in AD patients who are in an older cohort and have coincident morbidities, blood pressure should be monitored in order to reduce the adverse effects.

Another contributing mechanism to AD is mitochondrial dysfunction, which is a part of the disruption of cell bioenergetics in neurodegenerative diseases [40]. The AD-linked NMDA receptor (calcium channel) excitotoxicity causes mitochondrial dysfunction due to calcium overload [41]. Therefore, the NMDA inhibition action of ketamine may restore calcium hemostasis.

Neuroinflammation might play a key role in the initiation and progression of AD pathology [42]. Ketamine plays different roles to prevent exacerbation of inflammation, including regulation of the production of proinflammatory mediators and recruitment of inflammatory cells [43]. Furthermore, ketamine poses anti-apoptotic and antioxidant effects through the PI3K/AKT/GSK-3β pathway, which boosts its neuroprotective efficacy [4].

## 5. Recent Clinical Findings

There is a plethora of studies out there regarding ketamine and psychiatric disorders. The studies included in this section look at the role of ketamine in cognition, remission of psychiatric symptoms, and pre-clinical studies looking at animal models. A pre-clinical study by Zhang Ke et al. conducted a pre-clinical trial to elucidate the difference between the underlying molecular effects of ketamine and memantine that make the former one more susceptible to generating antidepressant effects. The authors used rats and looked at slices of their hippocampus and analyzed antibody staining of the studied protein via western blot. The study found that only ketamine induces the activation of mTOR and GluA1 expression, thus enhancing the excitatory synaptic transmission and inducing an anti-depressant action [44].

A 2021 randomized controlled trial investigated the efficacy of ketamine in patients suffering from chronic post-traumatic stress disorder (PTSD). This trial was performed after the authors’ proof-of-concept trial, which showed a reduction of PTSD symptoms after ketamine infusion after 24 h. In this study, 30 subjects with PTSD were randomized in a 1:1 ratio to receive six infusions of ketamine (0.5 mg/kg) or midazolam (0.045 mg/kg), which was used as a psychoactive control, over a two-week period. The authors found that 70% of individuals responded with significant improvement in their symptoms during this period as measured by their clinician-administered PTSD Scale for the DSM-5 from baseline [45].

In both AD and depression, problems with cognitive processing and memory are seen. With increasing evidence regarding ketamine’s effectiveness, prior studies highlighting its side effect of reduced cognitive function and problems with memory have been evaluated by multiple studies. Morgan et al. accessed the cognitive functions and behavioral activity in 18 heavy ketamine users (defined as self-administering ketamine three times a week) over the past year and compared them to 18 controls. The authors found a decrease in spatial memory with dysfunction in the hippocampus region of the brain, as seen on fMRI during neurocognitive tests [46]. On the contrary, a study by Murrough et al. involved the use of a randomized controlled trial to access the neurocognitive effects after infusion of 0.5 mg/kg ketamine amongst 65 depressed patients, and found a significant decrease in their condition with no cognitive impairment [47]. Likewise, a study by Davis et al., accessed the acute cognitive effects of ketamine with a single intravenous infusion and found no association with cognitive impairment or working memory performance [48]. Multiple studies show that minimal usage of ketamine at a controlled rate in accordance with the person’s tolerability induces no cognitive impairment [49].

A few studies have also reported improvements in processing speed, verbal memory, visual memory, working memory, and cognitive flexibility when treated with ketamine [50]. A 2020 study by Basso et al., compared the antidepressant and neurocognitive effects of ketamine to electroconvulsive therapy (ECT) when given to depressed patients. Although ketamine and ECT were equally effective, ketamine was rapid in terms of the onset of action and also showed improved neurocognitive behavior, while ECT showed a decline in the subjects’ cognition [51].

Likewise, a study by Zheng et al., conducted a clinical trial to access the neurocognitive functions among 64 depressed patients when treated with six intravenous infusions of 0.5 mg/kg ketamine over a 12-day period and followed by a two-week observational period. Four domains of neurocognitive function were assessed, which included the speed of processing, working memory, visual learning, and verbal learning. The study found improved cognitive functioning, especially with the speed of processing and verbal learning [52].

A 2021 randomized, double-blind placebo-controlled study aimed to access the efficacy of haloperidol and ketamine to prevent postoperative delirium. The study investigated the drugs separately and in combined form in comparison with the controlled group amongst 182 patients. The study found no prevention of cognitive decline postoperatively [53]. However, the study also found a significant decline of inflammatory markers with the use of ketamine postoperatively compared with the placebo group, suggesting ketamine’s neuroprotective action on neurons, glial cells, and astrocytes [53]. With studies showing increased levels of the neurotoxin quinolinic acid (QUIN), increased extracellular NMDA receptor agonists, and increased neuronal and glial cell death associated with AD, ketamine with opposing actions could be a practical approach for AD treatment [54]. Shibakawa et al., also studied the effect of ketamine on neuroinflammation and found that ketamine reduces the inflammatory cytokines affecting glial cells and astrocytes [55]. Thus, it indicated a profound association of ketamine with neuroprotection that could possibly have a therapeutic benefit in AD patients. These studies are summarized in Table 1.

## 6. Conclusions

Although there is currently no cure for AD, there are several approved medications and targets for drug therapy in clinical trials for symptomatic treatment. The FDA-approved medications for AD are acetylcholinesterase inhibitors and NMDA antagonists. Recent clinical findings in the last twenty years suggest that the nonselective NMDA antagonist ketamine may be beneficial in providing both neuroprotection and reduction of the neuropsychiatric symptoms in AD. Ketamine may prove to be more beneficial to patients than the standard treatments for AD because it has fewer side effects than acetylcholinesterase inhibitors and more of a broad mechanism of action than the NMDA antagonist, memantine. As a well-known analgesic and anti-inflammatory drug, it acts quickly, has long-lasting effects, and improves psychiatric symptoms with a smaller therapeutic dose than other medications. Ketamine has already proven successful in the treatment of psychiatric symptoms, specifically for treatment-resistant depression. This is important because depression may occur prior to memory loss in AD. In addition, ketamine is less likely to worsen cognition compared to other treatments like ECT for severe depression. Clinical trials have demonstrated that periodic doses of ketamine reduce symptoms such as suicidal ideation and psychosis. However, ketamine can also cause side effects such as dissociation, memory loss, and confusion. Based on these known side effects, the effect on individuals with dementia and depression may simply be explained as a drug side effect rather than a definitive improvement in symptoms. Additionally, vitals should be monitored closely when treating individuals with ketamine because individuals may experience an increase in blood pressure. This is a factor to consider before treating individuals with ketamine, especially in older individuals with cardiac comorbidities. Ketamine has been shown to act on the cellular level in opposing neurotoxins and protecting glial and neuronal function from the deleterious effects of inflammatory cytokines. Based on present information, we can summarize that ketamine may have a role in neuroprotection and the improvement of psychiatric symptoms in AD. Further research and clinical trials are warranted to prove or disprove this theory, but it is well worth investigating for a potential chance at improving the quality of life for millions of individuals.

## Figures and Tables

**Table 1:** Clinical Efficacy and Safety.

Author (Year)	Groups Studied and Intervention	Results and Findings	Conclusions
Study 1: [2]	Psychiatric (*n* = 29; 15 PTSD, 14 MDD) and healthy control group (*n* = 29) groups were recruited. General inclusion criteria: Adults 18–60 years old, no recent regular history of psychiatric medication use. Psychiatric group with MDD and/or PTSD, experiencing current major depressive episode (those with MDD), no other psychiatric conditions (except anxiety disorders), and free of psychotropic medications for at least two months (at time of ketamine injection). Racemic ketamine was administered to half the subjects in each group with an initial bolus of 0.23 mg/kg over 1 min followed by a constant infusion of 0.58 mg/kg per hour over 1 h. The remaining half received constant IV infusion 0.5 mg/kg ketamine over 40 min.	MDD/PTSD individuals showed significant improvement in the severity of depressive symptoms at both 2-h and one-day post-ketamine administration (severity decreased from moderate depression at baseline to mild depression). IV ketamine-induced declines in attention (ATTN), executive function (EF), and verbal memory (VM) 2 h post-administration, all resolved by one-day post-ketamine across groups. Degree of cognitive decline is larger in MDD/PTSD relative to HC solely on attention. No effect on working memory (WM) performance in either group.	Intravenous subanesthetic doses of ketamine have shown reduced psychiatric distress in both MDD and PTSD. However, it has an acute transient deleterious effect on cognitive domains in both MDD/PTSD and HC (most notably attention). The effect of ketamine on cognitive function in these disorders remains poorly understood.
Study 2: [3]	Adults 21–80 years of age with major depressive disorder (MDD) with a poor response to at least three therapeutic trials of an antidepressant. A single IV infusion of ketamine (0.5 mg/kg) or midazolam (0.045 mg/kg) over 40 min is infused. Neurocognitive tests were done.	At day seven post-treatment, improvement in processing speed, verbal learning, and visual learning from the baseline was noticed. ketamine responders had significantly slower processing speed at baseline than ketamine non-responders.	Processing speed, verbal learning, and visual learning improved at the end of the study after receiving ketamine compared with baseline. A rapid antidepressant effect at 24 h following ketamine is obtained with slower processing time at baseline.
Study 3: [4]	Inclusion criteria: Adults ranging from 18–65 years old, at least moderate depression (Montgomery-Åsberg Depression Rating Scale (MADRS) total score ≥ 25), evidence of treatment resistance, and secondary analysis baseline MADRS-SI suicide item score ≥ 2. A three-phase trial was conducted to assess the antidepressant effects of ketamine in treatment-resistant depression (TRD). Ketamine hydrochloride (0.5 mg/kg, diluted in 0.9% saline) and midazolam (30 μg/kg diluted in saline) infused over a 40 min. Separation of each infusion was by at least seven days. Followed up, infusion of six open-label ketamine thrice-weekly over two weeks. Participants showing antidepressant response (≥50% decrease in MADRS total score from baseline) of ketamine following	Ketamine MADRS-SI scores were lower than that of midazolam at 2 h and seven days post-infusion. Maximal effect of ketamine on SI measured at seven days post-infusion (mean decrease 1.7 points). At start of Phase 2, MADRS-SI scores were significantly lower than study baseline. Estimated mean MADRS-SI score for participants at post-Phase 2 follow-up visit was 1.0. with estimated mean reduction of 2.3 points on the MADRS-SI at end of Phase 2 from baseline.	Compared with midazolam, a single ketamine infusion elicited larger reduction in SI, with maximal effects measured at seven days post-infusion. 69% of participants had a complete alleviation of SI following repeated infusions. In TRD, single and repeated ketamine infusions resulted in decreases in SI which were maintained with once-weekly maintenance infusions.
Study 4: Ketamine vs. midazolam [5]	Adults (*n* = 41), ranging from 18–65, with treatment-resistant depression. Inclusion criteria: ≥25 on the Montgomery-Åsberg Depression Rating Scale (MADRS). Participants completed a single-site randomized double-blind crossover comparison of single infusions of ketamine and midazolam. After relapse of depressive symptoms, participants received a course of six open-label ketamine infusions administered thrice weekly over two weeks. Responders (participants with ≥50% decrease in their scores on MADRS) received four additional infusions administered once weekly (maintenance phase).	A single ketamine infusion elicited a significantly greater reduction in depressive symptoms at the primary efficacy endpoint compared with midazolam (24 h post-infusion). 59% of participants met response criteria after repeated infusions (median of three infusions needed before achieving response). Participants had no further change in MADRS scores during weekly maintenance infusions.	Repeated ketamine infusions have cumulative and sustained antidepressant effects that were maintained among responders through once-weekly infusions. Future studies should further expand on optimizing administration of ketamine in clinical settings, especially those associated with patients who suffer from treatment-resistant depression.
Study 5: Ketamine vs. ECT [6]	Fifty patients suffering from depression were treated with either serial ketamine infusions or ECT. Depression severity and cognitive functions were assessed before, during, and after treatment.	ECT and ketamine administration were equally effective. However, the antidepressant effects of ketamine occurred faster. Ketamine improved neurocognitive functioning, especially attention and executive functions, whereas ECT was related to a small overall decrease in cognitive performance.	Related to its pro-cognitive effects and faster antidepressant effect, serial ketamine administration might be a more favorable short-term treatment option than ECT.
Study 6: Ketamine vs. Haloperidol [7]	Pre-anesthetic, pharmacologic prevention of postoperative brain dysfunction with haloperidol, ketamine, and the combination of both vs. placebo in 182 patients.	None of the three pharmacologic interventions (haloperidol, ketamine, or both drugs combined) was significantly superior to placebo for preventing postoperative brain dysfunction and delirium. Measured levels of postoperative cortisol were significantly higher in delirious patients.	The study results offer no opportunity for an unprecedented option for preventing postoperative cognitive decline including delirium.

## Data Availability

All data listed in this manuscript is publically available from manuscripts found on PubMed.

## Abbreviations

AD	Alzheimer’s disease
NMDA	N-methyl-D-aspartate
SSRI	selective serotonin reuptake inhibitor
mTOR	Mammalian target of rapamycin
BDNF	Brain-derived neurotrophic factor
GluA1	Glutamate A1
SI	Suicidal Ideation
PTSD	Post-traumatic stress disorder
ECT	Electroconvulsive therapy
QUIN	Quinolinic acid
